# Heat Perception and Coping Strategies: A Structured Interview-Based Study of Elderly People in Cologne, Germany

**DOI:** 10.3390/ijerph18147495

**Published:** 2021-07-14

**Authors:** Juliane Kemen, Silvia Schäffer-Gemein, Johanna Grünewald, Thomas Kistemann

**Affiliations:** 1 GeoHealth Centre, Institute for Hygiene and Public Health, University Hospital Bonn, Venusberg-Campus 1, 53127 Bonn, Germany; silvia.schaeffer@ukbonn.de (S.S.-G.); thomas.kistemann@ukbonn.de (T.K.); 2 Environmental Planning and Prevention, The Environmental and Consumer Protection Office of Cologne City Council, Willy-Brandt-Platz 2, 50679 Köln, Germany; johannagruenewald@hotmail.com; 3 Department of Environment and Sustainability, Division of Climate Adaptation and Sustainable Land Use, German Aerospace Center Project Management Agency, Heinrich-Konen-Straße 1, 53227 Bonn, Germany; 4 Department of Geography, University of Bonn, Meckenheimer Allee 166, 53115 Bonn, Germany; 5 Center for Development Research, University of Bonn, Genscherallee 3, 53113 Bonn, Germany

**Keywords:** climate change adaptation, health, community health, heat perception, coping strategies, heat-health action plan, heatwave, self-reported health, climate change adaptation

## Abstract

The transdisciplinary project “Heat-Health Action Plan for Elderly People in Cologne” addresses the most heat-vulnerable risk group, people over 65 years of age. A quantitative study aimed to better understand heat perception and coping strategies of elderly people during heat waves to inform heat-health action plans. We conducted a representative quantitative survey via structured interviews with 258 randomly chosen people over 65 years old, living in their own homes in four areas of Cologne, Germany. These areas varied, both in terms of social status and heat strain. Data regarding demographics, health status, coping strategies, and heat perception were collected in personal interviews from August to October 2019. The majority of the participants perceived heat strain as moderate to very challenging. Women, people with a lower monthly income, and those with a lower health status found the heat more challenging. We found that participants adapted to heat with a number of body-related, home-protective, and activity-related coping strategies. The number of coping strategies was associated with perceived personal heat strain. There is a definite underuse of water-related heat adaption strategies among the elderly. This is of increasing relevance, as rising heat impact will lead to more heat-related geriatric morbidity. Our results are seminal to inform elderly-specific, socio-adapted local heat-health action plans.

## 1. Introduction

Rising global temperatures, with the last decade as the warmest on record, have contributed to increased and more intense extreme weather events [1]. Heat extremes are occurring with higher frequency and longer duration and are setting new temperature records [2,3]. In June and July 2019, the months before the study performance, Western Europe experienced two extreme heatwaves, with temperature records in several countries, including Germany. In Germany the historical record of 40.3 °C was broken at many stations, one of them Cologne-Stammheim (41.1 °C) by 1–2 degrees [4,5]. The next year, 2020, was the second warmest year registered ever, with a mean temperature of 10.4 °C (mean 1900–2000: 8.3 °C) and the third year of drought in a row on record [6].

Climate change threatens the very foundations of human health and wellbeing, with climate change as one of the five most damaging or probable global risks for the past decade [7]. Vulnerable populations experienced additional exposures to 475 million heatwave events, respectively 2.9 billion additional days of heatwaves globally in 2019 [8]. Heatwaves enhanced impacts to public health, especially for vulnerable groups like elderly people, are of additional significance, as the world’s population is aging rapidly [9,10]. Elderly people are the most vulnerable group because ageing brings about physiological changes that affect people’s thermal sensitivity and thermoregulation; moreover, there is a progressive increase of multimorbidity with increasing age [11]. Multimorbidity includes diabetes, cardiovascular, and respiratory diseases [12]. Additionally, increased social isolation has been identified as a risk factor [13]; this leads to excess morbidity and mortality. Excess mortality was 33% in those aged 75 and over, compared with 13.5% in those aged under 75 years [14]. During the past 20 years, there has been a 53.7% increase in heat-related mortality in people older than 65 years, with a global total of 296,000 such deaths in 2018 [8]. More than 70,000 additional deaths occurred in Europe during the heatwave in the summer of 2003 [15], 7000 of which were in Germany [16].

Aspects of demographics, pre-existing conditions, and the urban heat island effect lead to increasing health problems associated with heat. The demographic change shows a global distribution of the change in the percentage of people over 65 between 1980 and 2018, highlighting an increase of over 10% of this vulnerable group in some regions of Europe [10]. In the 65–74 age group, the risk of dying during heatwaves was higher among unmarried people and those with a previous hospitalization for chronic pulmonary disease or psychiatric disorders. In the age group 75 and older, women and unmarried people were more susceptible to heat. Furthermore, a higher susceptibility to heat among those with previous hospitalization for diabetes, diseases of the central nervous system (CNS), psychiatric disorders and cerebrovascular diseases related to these pre-existing conditions [17]. Other research with a general population underlines the higher mortality risk for women [18,19,20], widows or widowers [18], and people living alone [21,22,23]. Further socio-economic risk factors are lower education and lower income [18] or poverty [24]. However, it is not confirmed consistently that these socio-economic factors increase the risk of mortality [25,26]. Regarding housing conditions, non-access to functioning air conditioning is seen as a major risk factor as confirmed by many studies [21,27,28] as well as living in the top floor [21,29] or living in a flat instead of a house [28]. Health-related factors increasing the risk of mortality are fewer social or physical activities [27,30], hospitalization during the past two years [26,27], mobility restrictions [30], frailty [29], and several chronic or acute diseases such as diabetes, cardiovascular, pulmonary, and psychiatric diseases [21,23,28,31,32].

Another challenging aspect is that in many urban areas with high population density, climate change aggravates the so-called “urban heat island” effect: the high density of buildings, with reduced wind velocity and albedo, contributes to higher temperatures during days as well as nights in urban, as compared to rural, areas [31]. Considering that more than 50% of the world’s population currently resides in cities, with numbers continuing to increase rapidly, urban heat affects the majority of humans [33]. In Germany nearly 64 million (76%) live in urban settlements. These urban agglomerations include 79 cities with more than 100,000 inhabitants [34]. Cologne is one of four German cities with more than one million inhabitants. Due to its demographic factor distribution, ageing population, high rates of urbanization and prevalence of multimorbidity, the population of Germany tends to be increasingly at risk and highly vulnerable to heat exposure [12]. Effects tend to be unequal in different socioeconomic groups [8]. A survey of the general population in Germany indicated that 59% of those interviewed quote climate change as one of the biggest challenges (total Europe 47%) [35]. Against this background the German Federal Government adopted the „German Strategy for Adaptation to Climate Change” (Deutsche Anpassungsstrategie DAS) in 2008. This strategy aims at reducing the vulnerability of the population, the economy and the environment, and encourages the development of adaption capacities. Furthermore, the awareness of climate change and its consequences is to be promoted and relevant actors made aware of the significance of their own actions [36]. “Heat-Health Action Plan for Elderly People in Cologne” (2019–2021) is one of the lighthouse projects under the DAS umbrella. This project applies a transdisciplinary approach which combines public health research with administrative capacities at a local government level and the facilities of a regional water supplier. The project is conducted in Cologne, Germany, and is based on both qualitative and quantitative research methods. Two quantitative surveys were conducted, addressing on the one hand retirement home managers and, on the other hand, community-dwelling older adults in four areas of Cologne, targeting the population with an age of 65 years and older. Both approaches addressed knowledge, information flow, heat perception, coping strategies, and health aspects. This paper is focused on the elderly adults living independently as to their heat perception and coping strategies.

Several studies have been focusing on heat perception and coping strategies [37,38,39,40]. Few studies are focusing on the vulnerable group of elderly people [41,42,43]. These studies identified a variety of coping strategies during heatwaves. Banwell et al. [41] found that the participants reported a number of coping strategies including a universal use of air conditioning, adaptation of daily activity and changing dietary habits. The use of cooling appliances as a main strategy is underlined by the findings of Kondo et al. [42] and Nitschke et al. [43], in which the efficacy of targeted information was explored. They found an increased use of cooling systems after receiving information leaflets. Research in Germany on perception and coping strategies of an elderly population during episodes of heat is rare. There are only two studies focusing on heat-related behavior in older adults during heatwaves [44,45]: Lindemann et al. [44], examining a sample from sheltered care facilities, found that social participation decreases with increasing temperature whereas water intake increases; Conrad and Penger [45] showed the decreased mobility behavior during cold and hot episodes and presented a relation between health status and heat perception. Internationally, there has been limited research adding aspects of self-perceived health or participant’s health outcomes in general. Consequently, we integrated self-perceived health and a functional ability index based on a set of questions regarding health resources and health risks into our approach.

The objectives of our study, conducted within the DAS-project, were to assess and identify: (i) the heat perception and perception of vulnerability of an elderly population in Germany; (ii) coping strategies during episodes of heat used by an elderly population in Germany; (iii) adverse health effects of heat and the examination of heat related aspects of GP consultations; (iv) heat-related information sources and knowledge; (v) mobility aspects during episodes of heat; and (vi) social participation in general and during heat. In this paper we present the findings of objectives (i) and (ii). We intend to publish further results soon. Our assumptions were that heat perception is strongly influenced by health-related factors and that there is a lack of coping strategies. Our findings seek to improve heat-health risk communication in the context of heat-health action plans.

## 2. Materials and Methods

### 2.1. Study Areas and Data Collection

**Figure 1:**
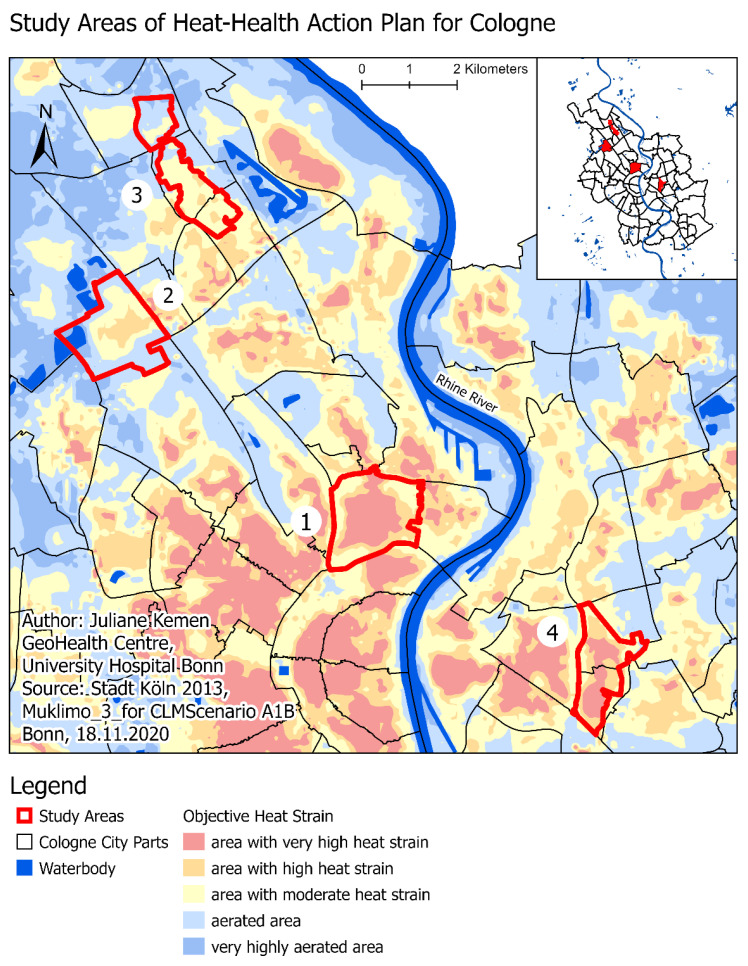
Study Areas of Heat-Health Action Plan in Cologne.

A quantitative survey was conducted with community-dwelling adults above the age of 65 years from August up through October 2019 in four urban areas of Cologne. A pre-test was conducted in July 2019. The sample included people living in these four areas (Figure 1). The study areas were chosen to depict the range of both socioeconomic and climatic living conditions in the city. Therefore, the selection of study areas was based on two dimensions: socioeconomic status (affluent vs. deprived) and objective heat impact (less impacted vs. highly impacted). Area 1 is characterized as being affluent and highly heat-impacted, area 2 as affluent and less heat-impacted, area 3 as deprived and less heat-impacted, and area 4 as deprived and highly heat-impacted (Figure 1).

Study areas 3 and 4 are so-called “social areas”, as defined by the city of Cologne, which are characterized by social deprivation above the city average [46]. Socio-economical (e.g., percentage of unemployed people, percentage of households with low income), political-cultural (e.g., percentage of non-voters, share of people with no cultural participation), and health-related (e.g., obese children, percentage of people with no sport-related activities) aspects of deprivation have been considered in the definition. Study areas 1 and 2 are city zones not designated as “social areas”, as they are populated by a more affluent population. The data of objective heat strain is based on Cologne’s climate data analysis conducted in a previously conducted project, “Klimawandelgerechte Metropole Köln” [47]. Figure 1 shows the predicted climatic characteristic in 2021–2050 for the urban area of Cologne. For instance, the (red) area with a very high heat strain is characterized by 10K warmer temperature during episodes of extensive heat as compared to the (orange) area with high heat strain and has a 40–80% higher number of days with a perceived temperature above 32 °C. To select areas with a higher or lower objective heat strain, we conducted a vulnerability analysis including these climate data and the share of elderly people living in social areas or city zones, respectively. Study areas 2 and 3 are both characterized by very few elderly people living in areas with very high or high heat strain (<30%). Study areas 1 and 4 are characterized by a high share of elderly people living in areas with a high or very high heat strain (>75%).

In each of the four study areas, we randomly selected 690 people from city council data aged 65 years and older. These 2760 older people received a municipal letter with a description of the study and a polite request to participate. A team of interviewers visited the areas from August to October 2019. We conducted face-to-face interviews with nearly 10% of 2760 addressed people living in private homes (*n* = 258 people). Table 1 shows the age groups of the total population in the study areas as compared to the number of contacted people and the number of people in the sample. In study areas 1 and 4, age class III is clearly underrepresented in the sample. All over the sample there is a lesser participation of older aged people. Otherwise the distribution of age groups is fairly similar to the full sample. The interviews lasted between 20 and 60 min. To record the answers, we used tablets and the online-survey tool “umfrage-online.de”. The interviewer team also provided printed questionnaires for those who did not want to participate in a face-to-face interview. To include people with an immigrant background, the questionnaire was translated into Russian and Turkish, as these two communities represent the largest non-German speaking ethnic groups in the study areas. All data were anonymized.

All subjects gave their written informed consent for inclusion before they participated in the study. The study was conducted in accordance with the Declaration of Helsinki. The protocol was approved by the Ethics Committee of the Medical Faculty of the University of Bonn (Project identification code: 265/19) and the data protection council of the University Hospital Bonn.

The topics of the standardized interview were developed based on a literature review and by drawing on the experience of the project’s scientific council. The main part of the interview was structured, whereas it contained some open questions (see Supplementary Word S1). The questionnaire covered several thematic areas, out of which heat perception, heat coping strategies, and self-reported health will be presented in this paper. We considered hot days as days with a maximum temperature over 30 °C and heatwaves as several hot days (>3 days) in a row without a cool night (tropical night) [48].

### 2.2. Interview Topics

#### 2.2.1. Demographic and Socioeconomic Characteristics

Demographic factors included sex, age, education, former occupation, house owner or not, living space, number of people in household, and household income per month.

#### 2.2.2. Perceived Heat Strain

Perceived heat strain and perceived health risk were measured on a 5-level Likert scale by asking “How would you rate your personal heat strain?” and “How would you rate your risk for personal health issues during heatwaves?”.

#### 2.2.3. Coping Strategies

Adapted heat coping strategies were explored through the question “What do you do during an episode of heat to protect yourself?” as well as several more detailed questions addressing specific adaptation measures, including air conditioning (AC), ventilation, dressing more lightly, using water to cool down and the rescheduling of activities. We also asked about access to AC and shading options like blinds, shutters, and curtains.

#### 2.2.4. Self-Reported Health and Functional Ability

Self-reported health was measured through the question “How is your health in general” with five possible answers (very good—very poor) [49]. Health status was also measured by using the self-reporting Functional Ability Index (FA Index) which is based on twelve questions regarding health risks and resources. This index was primary developed to screen functional competence of older adults in community settings [50]. Based on these variables, a score is built which classifies the participants into four groups: ROBUST (=many resources and few risks), postROBUST (=many resources and many risks), preFRAIL (=few resources and few risks) and FRAIL (=few resources and many risks). As health risk indicators, the index considers: loss of weight (>5 kg) during the last six months, degradation of walking, climbing of steps or the ability to get into a car or bus, furthermore a falling event during the last 12 months. As health resources, the index considers: ability to walk 500 m without difficulties or medical accessories (e.g., walking stick or rollator), participating at sport activities, or community service and more than two days during the week spending time outside the house or flat. Only people without daily need of assistance or professional care were included into the variable [50]. The index has been used in several publications on frailty, functional decline, and disability in the setting of community-dwelling older adults [51,52].

### 2.3. Statistical Analysis

Statistical analysis was conducted by use of IBM SPSS Statistics V25 (International Business Machines Corporation, Armonk, NY, USA). We applied Chi^2^-Tests, Kruskal-Wallis-Tests, and Mann-Whitney-U-Tests to test for significant differences between groups. We used Spearman correlation to calculate the direction and strength of correlations.

## 3. Results

### 3.1. Demographic, Socio-Economic, and Health-Related Characteristics of Participants

A total of 258 people were interviewed from August through October 2019: 69 people in study area 1; 69 people in study area 2; 59 people in study area 3; and 61 people in study area 4. The age of the participants was between 65 and 93 years. Table 2 displays the sociodemographic characteristics of the participants. Women more frequently live alone and live in households with a lower monthly income. Women also have lower level school leaving certificates, are more often housewives or manual workers, and are rarely in a managing position. More than twice the number of men than women hold an academic degree (37.0% vs. 15.3%). We did not find any significant differences between men and women respecting age classes, self-reported health status, FA Index, and social contacts.

### 3.2. Perception of Heat Strain and Vulnerability

The participants were asked how they perceived their personal heat strain during a heat episode. About a quarter of them reported perceiving not any (*n* = 22; 8.6%) or a little (43; 16.6%) heat strain (*n* = 256). Most participants reported perceiving a moderate heat strain (110; 42.6%). One third rated the heat clearly (51; 19.9%) or extremely challenging (30; 11.7%). We identified significant differences for gender, former occupation, household income, self-reported health status, FA Index, and household size (Table 3). The results suggest that women tend to find heat more challenging. Self-employed people, employees, and public servants in a managing position found the heat very challenging less frequently.

For ordinal variables which showed significant differences (self-reported health status, FA Index, household income), a Spearman correlation coefficient was calculated. People with a lower self-reported health status experienced heat as more challenging (Spearman’s ρ = 0.397, *p* = 0.000, *n* = 252). Additionally, people with a higher FA Index (i.e., fewer resources and more risks) reported more heat strain (Spearman’s ρ = 0.217, *p* = 0.001, *n* = 226). The more household income a person declared the less heat strain was perceived (Spearman’s ρ = −0.189, *p* = 0.008, *n* = 198).

We could not detect any differences between people living in areas with a relatively high objective heat strain (area 1 and 4) as compared to those living in areas with relatively low objective heat strain (areas 2 and 3). The same was true for people living in houses as compared to people living in a flat. Likewise, age, school leaving certificate, and social contacts did not show a significant correlation for our sample.

The participants were asked for reasons why they perceived the heat strain as being not challenging or less challenging, respectively moderate, clearly or very challenging. Reasons were categorized afterwards. Subjective reasons why participants (*n* = 59) perceived no or little heat strain were “less heat sensitivity” (24; 40.7%), “adaptive strategies” (22; 37.3%), “good health status” (13; 22.0%), “enjoying heat” (8; 13.6%), or other reasons (3; 5.1%). Reasons for a moderate to extremely challenging heat perception (*n* = 177) were categorized into six categories. Thirty-three people mentioned a “general heat sensitivity” (18.6%), 46 specified “co-morbidities or a poor health status” (46.3%). For some of the participants it was “age” per se what made them suffer from heat (15; 8.5%). “Mobility constraints or reduced social interactions” were also mentioned several times (18; 10.2%).

We found a similar distribution for the perceived risk to suffer from personal health issues during heatwaves. Around 40% of the participants (*n* = 251) assessed this risk to be very low (34; 13.5%) or low (66; 26.3%). While slightly more than a third rated the risk as moderate (92; 36.7%), the rest thought it would be high (43; 17.1%) or very high (16; 6.4%). 

When we were asking with an open question about vulnerability to heat strain of different population groups, most commonly, “older people” were seen to be vulnerable (217; 84.1%). Children (121; 46.9%), people with diseases (117; 45.3%), babies (80; 32.9%), outside workers (27; 10.5%), pregnant women (8; 3.1%), people with limited mobility (7; 2.7%), outside athletes (2; 0.8%), and overweight people (2; 0.8%) were mentioned as being vulnerable as well.

### 3.3. Heat Coping Strategies

Based on literature we identified three different groups of heat coping strategies: body-related strategies (wearing less or thinner clothes, drinking more fluids, eating differently, taking showers more frequently, cooling arms with water, using wet towels, cooling feet with water), home-protective strategies (using thinner beddings, opening windows for ventilation, closing blinds/shutters, turning on a fan and AC), and activity-related strategies (reducing and rescheduling physical activities) [53]. The majority of respondents used multiple approaches for heat-coping strategies (Table 4). Use of electric devices, such as fans or AC, was uncustomary. While nearly half of the participants (47.3%) stated that they used a fan, it was uncustomary to use AC (3.9%) on hot days. Water-related cooling strategies such as “Cooling arms with water”, “Cooling feet with water”, and “Using wet towel” were used only by a few people. The number of heat-coping strategies varied between 2 and 13, while the majority combined a set of 8–10 strategies.

Regarding the access to AC we found that the number of people owning AC is identical with the number of people using AC (10; 3.9%). One person stated to use AC permanently during hot days, six people would use it for several hours a day, and three people claimed to use it very rarely.

Regarding the access to shading options we found that around two thirds of the participants (154; 61.4%) owned rolling-shutters, almost half of the sample had awnings (115; 45.8%) or curtains (113; 45.0%), others owned blinds (94; 37.5%) or folding-shutters outside the building (6; 2.4%). Every participant had at least one shading option in his flat or house (1 option: 89; 35.5%; 2 options: 106; 42.2%; 3 options: 44; 17.5%; 4 options: 11; 4.4%; 5 options: 1; 0.4%).

### 3.4. Correlation of Coping-Strategies and Perception of Heat Strain, Socio-Economic, and Health-Related Factors

The sum of all coping-strategies is positively correlated with perception of heat strain (Spearman’s ρ = 0.396 **, *p* = 0.000, *n* = 256). More adapted coping strategies are associated with more perceived heat strain. We also found positive correlations of the sum of body-related strategies (Spearman’s ρ = 0.328 ***, *p* = 0.000, *n* = 256), of home-protective strategies (Spearman’s ρ = 0.208 **, *p* = 0.001, *n* = 256), as well as of activity-related strategies (Spearman’s ρ = 0.285 **, *p* = 0.000, *n* = 256) with perception of heat strain.

We used a Chi-Square Test to demonstrate which of our demographic or further reasonable variables had an influence on each coping strategy (Table 5, Table 6 and Table 7). We excluded wearing less or thinner clothes from that analysis as it was used by 99.2% of the participants. Looking at gender, we found statistically significant different usage patterns regarding thin bedding and cooling arms with water: 88.0% of the men (*n* = 111) and 96.2% of the women (*n* = 126) stated using thinner bedding; 38.2% of the women (*n* = 50) stated that they would cool their arms with water, whereas only 22.0% of the men (*n* = 28) reported doing so.

With respect to age, we found differences in eating differently and showering more frequently: with increasing age, participants changed their diet less frequently during heatwaves (Age Class I: 116, 81.7%, Age Class II: 69, 73.4%, Age Group III: 10, 47.6%). The distribution was similar for the frequency of taking a shower (Age Class I: 107; 75.4%, Age Class II: 66; 69.5%, Age Class III: 10; 47.6%).

Level of education showed differences concerning fluid consumption habits on hot days. People with lower-level school leaving certificates increased their water intake less frequently during hot days as compared to people with higher-level certificates. The participants with the highest school leaving certificates (group I: 78, 87.6%) drank more water most frequently. The percentage decreased to its lowest in the group without any school leaving certificate (group II: 34, 82.9%, group III: 83, 77.6%, group IV: 1, 33.3%).

Household income per month was correlated with strategies such as cooling arms with water, using wet towels and using AC. Cooling arms with water was more often used in groups with less income. We found the rarest use of this strategy in participants with a household income of 3000 Euro or more (I: 7, 43.8%, II: 24, 33.3%, III: 20, 41.7%, IV: 11, 17.7%).

Lower health status was identified with cooling arms with water more often. From the participants with the lowest health status, 85.7% claimed to cool their arms with water, whereas people with a better health status used this strategy less frequently (health status very good: 4, 20.0%, good: 24, 22.6%, fair: 35, 36.1%, poor: 9, 39.1%, very poor: 6, 85.7%). Participants with a fair to very poor health status always used the strategy of closing blinds, shutters, or curtains (very good: 16, 88.9%, good: 88, 98.9%, fair: 90, 100%, poor: 17, 100.0%, very poor: 7, 100.0%). Lower FA Index went along with decreased number of people showering more frequently (ROBUST: 118, 72.0%, postROBUST: 29, 87.9%, preFRAIL: 19, 67.9%, FRAIL: 17, 51.5%).

The more social contacts the participants had, the more they opened windows for ventilation (one or more social contacts per week: 210, 99.1%, two to three social contacts per month: 20, 100.0%, one social contact per month: 7, 77.8%, more seldom or never: 12, 85.7%).

Household size turned out to have an influence on fluid consumption and use of AC. Fewer singles drank fluids as frequently during episodes of heat than participants living together with at least one person (single: 66; 72.5%, two people: 129; 87.2%, more than two people: 10; 83.3%). Non-single households were more likely to own AC (single: 1; 1.1%, two people: 7; 4.7%, more than two people: 2; 16.7%).

Participants living in areas with higher heat strain used wet towels to cool down more frequently (objectively higher heat strain: 28, 29.2%, objectively lower heat strain: 22, 17.2%).

People living in houses as compared to flats more often owned AC (flat: 3; 1.8%, house: 7; 8.0%).

There were no discernible differences in coping strategies with respect to level of education and GP consultation for medical advice about heat adaption strategies.

## 4. Discussion

### 4.1. Perception of Heat Strain and Vulnerability

Elderly people are a vulnerable group during heatwaves, as previous research shows [15,54]. We conducted a quantitative study to better understand the perception of heat strain and coping strategies of the elderly. As experimentally demonstrated, perceived heat is strongly associated with physiological thermal strain [55]. Against that background, and as no other method was available, we used perception of heat strain as adequate proxy to assess thermal heat stress. We found that the majority of the participants reported finding the heat moderately or clearly challenging. This goes along with findings of Kalkstein and Sheridan [56] who conducted a survey in Phoenix, Arizona, USA, focusing on heat perception and behavioral changes as a result of heat warnings. To measure the perceived risk, they asked 201 participants: “How dangerous do you think the heat is for you?” Two thirds of their participants perceived heat as being “somewhat dangerous” (25.0%) to “very dangerous” (44.7%). Only 7.6% rated heat as “not at all dangerous”. Our findings concerning women tending to find heat more dangerous are consistent with that study. A study in Southern Germany with independent older adults living in sheltered care facilities revealed that of the 79 participants of a structured interview, 77.2% perceived heat waves as stressful or very stressful [44]. While few participants in a study with individuals aged 75 years or older in London and Norwich, UK, found heat extremely challenging, many reported feeling uncomfortable [53]. Liu et al. [39] uncovered a lower risk perception in males, less educated people, people who had extremely low income or extremely high income, and people employed in agriculture, forestry, animal husbandry, or fishing.

Our study showed heat perception as significantly correlated with both FA Index and self-reported health status, but not with age per se. The FA Index applied here was developed to screen functional competence of community-based older adults [50]. The FA Index distribution of our sample matches the large primary study conducted in Hamburg, Germany (since 2001). FA Index correlates with self-reported health, chronic pain and depressive mood [50]. Dapp et al. [50] claim that age per se has minor significance in discriminating between robust, prefrail, frail and disabled people: “These classes show different perspectives of functional decline, developing need of nursing care and survival” [50]. There are few other studies addressing health status or functional ability in the context of heat perception and coping strategies. Our findings are in line with results of a telephone survey with 499 South Australians aged 65 and older who found themselves to be more at risk during extreme heat if they had reduced self-assessed health status (OR 2.3, 95% CI: 1.3, 4.4, *p* < 0.05) or used mobile aids (OR 2.2, 95% CI:1.1, 4.3 *p* < 0.05) [57]. Interestingly, many of our participants were aware of a link between their personal health status and their personal heat perception. Those who felt that heat was not particularly challenging often mentioned a “good health status” while those with stressing heat perception specified “co-morbidities or a poor health status” as reasons for their perception.

Considering vulnerability, our results show that almost 85% of the participants considered older people to be a vulnerable group during heat events. By contrast, we found that only around one fifth of the participants considered their personal risk of suffering from personal health issues during heatwaves as high (43; 17.1%) or very high (16; 6.4%). Thus, we can confirm findings from London and Norwich, UK, revealing that only a few respondents considered themselves to be elderly or at risk in episodes of hot weather, although the researchers of these findings classified all their participants as being vulnerable according to heat [53]. A quantitative study in Australia showed the same tendency of underrating the perception of personal risk of heat: people stated having no perception of heat as a threat as they “had lived with it all their lives” [41]. A study in New York, USA, with four focus-groups comprising a total of 38 participants (seniors aged >65 years or their care-givers) found that most seniors knew about the dangers of heat for older people but did not see themselves as being at risk [37]. All these findings, set out in this paragraph, underpin the conclusions of the studies that it is crucial to inform the public and particularly the elderly about heat risks.

### 4.2. Coping Strategies

Nine different heat coping strategies were adapted by more than 70% of the participants. Some strategies, e.g., water-related strategies such as cooling arms and feet with water or the use of wet towels are rarely used. A review showed that taking extra showers or baths was associated with a lower risk of death during a heatwave [32]. We assume based on underuse of these diverse strategies that the participants might not know about the positive effects of these strategies, and we clearly recommend public information about the positive outcomes. In France, a survey showed that the awareness of heat risk after heat alerts broadcast on radio and television was highly associated with an increase of coping strategies (e.g., hydration, closing sun-facing windows) from 6–15% [58].

Our results indicate that coping strategies are associated with the perception of personal heat strain. We recognized that people who found heat more challenging (higher heat perception) applied more coping strategies. This effect could be shown for body-related, for home-protective, and for activity-related coping strategies. Our results are in line with the work of Kalkstein and Sheridan [56] who found perceived risk to be a strong predictor as to whether or not an individual changed their behavior. Kalkstein and Sheridan [56] were also interested in how people reacted to heat warnings and which behavioral changes they showed. While more than 80% of the participants who perceived heat to be very dangerous for themselves changed their behavior, only 20% of those who found heat “not at all” dangerous performed behavioral adaptions. Women were slightly more likely than men to change behavior on heat warning days. We could confirm this gender effect for only two coping strategies, namely the use of thinner beddings and cooling arms with water.

Eighty percent of our participants stated that they would drink more fluids during hot days. Again, this finding is in line with the results of Kalkstein and Sheridan [56] who found that 83.3% of their participants reported drinking more fluids after receiving heat warnings. It is important to consider that people stating they would drink more fluids does not prove that they drink a sufficient amount of fluids. As reported elsewhere, another outcome of our study is that even on a day with moderate temperatures, only one third of the participants drank a sufficient amount of fluids [59]. 

Interestingly, participants’ use of electric fans (47.3%) is only moderate and use of AC is extremely low (3.9%). This seems to be a typical phenomenon of Northern or Western European countries: by way of example, in Vienna, Austria, only 7% of randomly selected participants of a survey with people 65 years of age and older had AC at home [60]. Many non-European studies show that for many elderly people, the use of AC is the main or even the only adaption strategy [41,42,53,61,62,63]. In Canada, only 2% of the Montreal residents with chronic cardiac and pulmonary disease did not use AC or a fan [63]. Lee and Shaman [61] found that use and frequency of AC in the bedroom were significantly associated with better thermal comfort for 706 survey participants in New York, NY, USA. In addition to thermal comfort, the use of AC is also associated with a decreased mortality risk during heatwaves [32]. Nonetheless, AC as a coping strategy has limits with regards to energy consumption and technical dependency. We did not ask the respondents in our sample why they would not own or use AC as a strategy against heat, but some authors suggest high energy cost might be one factor not to install AC or make only sporadic use of it, especially for low-income households [64,65]. With respect to climate change mitigation strategies we do not suggest the installation of AC for elderly people without a clear assessment of personal benefit and climatic disadvantages. In contrast, we found that it is very common to own and use shading options like curtains, shutters, and blinds. All of our participants had access to at least one shading option and 85.7% made use of them.

### 4.3. Limitations

We conducted face-to-face interviews. For some participants, this may have resulted in socially accommodating answers (social-desirability bias). Even though we randomly selected 2760 participants out of city council data and reached the targeted 10% of this sample, not every participant had an equal chance to be part of the sample (selection bias). As the interviewers walking on foot through the urban areas were small in number, they were not able to ring the doorbell more than once at every residence. Despite these limitations, conducting interviews face-to-face or via telephone are well-established methods of gathering information about a population [56]. Even though the study was only conducted in one city, through the variety of social characteristics and subjective heat impact it can be assumed that results are transferrable to other contexts. 

Reporting bias may have influenced the results of some variables, such as the daily fluid intake, the use of coping strategies, or self-reported health, as we did not measure them but ask for outcomes. We measured health through self-reported health status and the FA Index which is also based on self-reported factors. On the one hand, self-reported health data could be considered unreliable compared to objectively measured data; however, on the other hand, research has shown the predictive character of self-reported data [66,67].

## 5. Conclusions

Our study illustrates that the vast majority of elderly people surveyed are implementing at least some coping strategies against heat strain. Further questions into which strategies were applied showed that some strategies are underused. Suitable communication through varied media channels should be used to increase the implementation of mitigation measures. Encouragement of media consumption of heat warnings on a daily basis is essential, as this is the only way to raise sustainable awareness among the population. In order to ensure that recommended measures are carried out, these measures need to be adapted to the respective target group and incorporated into everyday routines.

Kalkstein and Sheridan [56] point out that coping strategies for heatwaves, although associated with an extreme increase of morbidity and mortality, might be less memorable than other natural catastrophes. According to these findings and to our own results, we strongly recommend extensive, targeted information campaigns for the vulnerable population. These should include but are not limited to short information leaflets or comprehensive information booklets. The project “Heat–Health Action Plan for Elderly People in Cologne” is using the short “Hitzeknigge” published by the German Environment Agency (UBA) and includes local information such as location of pharmacies, cooling centers, or drinking fountains [68]. An example for a comprehensive booklet is a brochure by Lindemann et al. [69] which includes target oriented information about adverse health effects through heat and provides detailed recommendations how to keep the apartment cool as well as the body cool and hydrated during episodes of heat. In addition, we recommend creative communication methods which remind readers on a playful or subliminal basis on the use of coping strategies. One humorous but still practical example is the catchy song “Drinke!” (Cologne idiom for “Drink!”), reminding elderly people to drink every 30 min, sung in the local idiom and published by a local band for the Cologne project. Finally, wearable sensors to measure humidity and temperature could raise heat perception of the vulnerable population and additionally would provide data for further research projects [70].

Mees et al. [71] revealed that cities still do not adequately address the protection of their vulnerable citizens against extreme heat. They suggest strong public intervention, as well as public–private partnerships for cooperation, during heatwaves to ensure that people living alone who are dependent on help due to social isolation are also taken into account. We emphasize these recommendations and invite cities to adapt towards heatwaves with centrally coordinated task forces with local stakeholders as well as health and climate experts. Representatives of vulnerable population groups need to be integrated into these task forces just as social workers, health insurances, local pharmacies, general practitioners, and civil protection are.

Heatwaves occurring in urban agglomerations are particularly challenging for the vulnerable group of the elderly related to their physiological changes and progressive increase of multimorbidity. Since 2020, this group is moreover one of the most vulnerable groups facing the COVID-19 pandemic challenge. The burden is twofold and self-reinforcing. Facemasks, for example, used to prevent spread of the SARS-CoV-2 virus, may aggravate cardio-pulmonary effects through heat strain. We underscore that Heat Health Action Plans are urgently needed to provide help towards assisting those people particularly at high risk adapt effective coping strategies at a time where the daily priority is to avoid potentially fatal SARS-CoV-2 infection.

## Figures and Tables

**Table 1:** Age classes of population in the study areas compared to sample (Age Class I: 65 to 74 years, Age Class II: 75 to 84 years, Age Class III: over 84 years).

Variables	Number of People in Study Area (%)	Number of Contacted People	Number of People in Sample (%)
Study Area 1			
Age Class I	2510 (50.2)	336	40 (58.0)
Age Class II	1898 (38.0)	255	25 (36.2)
Age Class III	588 (11.8)	79	4 (5.8)
Study Area 2			
Age Class I	1007 (44.5)	298	35 (50.7)
Age Class II	1034 (45.7)	306	27 (39.1)
Age Class III	219 (9.7)	65	7 (10.1)
Study Area 3			
Age Class I	2436 (52.1)	349	35 (59.3)
Age Class II	1713 (36.6)	245	18 (30.5)
Age Class III	527 (11.3)	76	6 (10.2)
Study Area 4			
Age Class I	2075 (49.9)	334	32 (52.5)
Age Class II	1568 (37.7)	253	25 (41.0)
Age Class III	512 (12.3)	82	4 (6.6)

**Table 2:** Sociodemographic statistics of the participants (* *p* < 0.05, ** *p* < 0.01, *** *p* < 0.001 Chi^2^-Test for comparison between men and women).

Variables	Men	Women	Chi-Quadrat	*p*-Value
	*n* (%)	*n* (%)		
Age: mean (range)	75 (65–93)	74 (65–93)		
Age classes (*n* = 258)	*n* = 127	*n* = 131		
I: 65 to 74 years	61 (48.0)	81 (61.8)	χ^2^(2) = 5.846	0.054
II: 75 to 84 years	56 (44.1)	39 (29.8)		
III: over 84 years	10 (7.9)	11 (8.4)		
Living situation (*n* = 257)	*n* = 126	*n* = 131		
alone	31 (24.6)	64 (48.9)	χ^2^(2) = 16.212	0.000 ***
with one other person	88 (69.8)	62 (67.3)		
with >2 people	7 (5.6)	5 (3.8)		
Monthly Household Income (*n* = 198)	*n* = 95	*n* = 103		
I (<1000 €)	7 (7.4))	9 (8.7)	χ^2^(4) = 23.727	0.000 ***
II (1000–<2000 €)	21 (22.1)	51 (49.5)		
III (2000–<3000 €)	29 (30.5)	19 (18.4)		
IV (≥3000 €)	38 (40.0)	24 (23.3)		
School leaving certificate (*n* = 245)	*n* = 121	*n* = 124		
I Academic secondary school	60 (49.6)	31 (25.0)	χ^2^(4) = 21.008	0.000 ***
II Secondary school	15 (12.4)	6 (21.0)		
III Secondary general school	46 (38.0)	60 (48.4)		
IV No school certificate	0 (0.0)	4 (3.2)		
Former Occupation (*n* = 250)	*n* = 123	*n* = 127		
Self-employed	13 (10.6)	8 (6.3)	χ^2^(5) = 35.353	0.000 ***
Employee or public servant in a managing position	43 (35.0)	19 (15.0)		
Employee or public servant				
Skilled worker	43 (35.0)	71 (55.9)		
Worker	21 (17.1)	9 (7.1)		
Farmer	3 (2.4)	13 (10.2)		
Housewife/homemaker	0 (0.0)	0 (0.0)		
	0 (0.0)	7 (5.5)		
Academic Education (*n* = 258)	*n* = 127	*n* = 131		
Yes	47 (37.0)	20 (15.3)	χ^2^(1) = 15.854	0.000 ***
No	80 (63.0)	111 (84.7)		
Self-reported health status (*n* = 226)	*n* = 124	*n* = 129		
Very good	10 (8.10)	10 (7.8)	χ^2^(4) = 3.423	0.49
Good	58 (46.80)	48 (37.2)		
Fair	43 (34.70)	54 (41.9)		
poor	11 (8.90)	12 (9.3)		
Very poor	2 (1.60)	5 (3.9)		
FA Index (*n* = 226)	*n* = 116	*n* = 110		
ROBUST	82 (70.7)	78 (70.9)	χ^2^(3) = 1	0.972
postROBUST	14 (12.1)	15 (13.6)		
preFRAIL	11 (9.6)	9 (8.2)		
FRAIL	9 (7.8)	8 (7.3)		
Social contacts (*n* = 256)	*n* = 125	*n* = 131		
Once or more a week	102 (81.6)	110 (84.0)	χ^2^(3) = 6.536	0.088
Two or three times a month	11 (8.8)	9 (6.9)		
Once per month	8 (6.4)	2 (1.5)		
Less than once per month	4 (3.2)	10 (7.6)		

**Table 3:** Perception of heat strain (* *p* < 0.05, ** *p* < 0.01, *** *p* < 0.001, Mann-Whitney U-Test, or Kruskal-Wallis-Test).

Variables	Perception of Personal Heat Strain						
	No	Little	Moderate	Clearly	Very Much	Mann-Whitney U-Test (W)	
	(%)	*n* (%)	*n* (%)	*n* (%)	*n* (%)	Kruskal-Wallis-Test (H)	
Gender							
Female	8 (6.1)	15 (11.5)	60 (45.8)	24 (18.3)	24 (18.3)	W = 14294	*p* = 0.002 **
Male	14 (11.2)	28 (22.4)	50 (40.0)	27 (21.6)	6 (4.8)		
Age Class							
I: 65–74 years	11 (7.8)	28 (19.9)	61 (43.3)	24 (17.0)	17 (12.1)	H = 1.260	*p* = 0.532
II: 75–84 years	8 (8.5)	13 (13.8)	39 (41.5)	22 (23.4)	12 (12.8)		
III: >84 years	3 (14.3)	2 (9.5)	10 (47.6)	5 (23.8)	1 (4.8)		
Household size							
single	10 (10.5)	10 (10.5)	37 (38.9)	20 (21.1)	11 (11.6)	H = 6.785	*p* = 0.034 *
with one other person	12 (8.1)	12 (8.1)	68 (45.6)	27 (18.1)	16 (10.7)		
with more than one person	0 (0.0)	0 (0.0)	4 (36.4)	4 (36.4)	3 (27.3)		
School leaving certificate							
I Academic secondary school	11 (12.1)	17 (18.7)	39 (42.9)	18 (19.8)	6 (6.6)	H = 7.283	*p* = 0.063
II Secondary school	5 (12.2)	6 (14.6)	20 (48.8)	9 (22.0)	1 (2.4)		
III Secondary general school	6 (5.5)	16 (14.7)	45 (41.3)	23 (21.1)	19 (17.4)		
IV No school certificate	0 (0.0)	1 (25.0)	2 (50)	0 (0.0)	1 (25.0)		
Former Occupation							
Self-employed	5 (23.8	3 (14.3)	5 (23.8)	7 (33.3)	1 (4.8)	H = 12.340	*p* = 0.030 *
Employee or public servant in a managing position	4 (6.5)	19 (30.6)	24 (38.7)	12 (19.4)	3 (4.8)		
Employee or public servant							
Skilled worker	8 (7.0)	16 (14.0)	56 (49.1)	19 (16.7)	15 (13.2)		
Worker	3 (10.3)	2 (6.9)	10 (34.5)	9 (31.0)	5 (17.2)		
Farmer	2 (12.5)	3 (18.8)	7 (43.8)	1 (6.3)	3 (18.8)		
Housewife/homemaker	0 (0.0)	0 (0.0)	0 (0.0)	0 (0.0)	0 (0.0)		
	0 (0.0)	0 (0.0)	2 (28.6)	3 (42.9)	2 (28.6)		
Household Income per month							
I (<1000 €)	3 (18.8)	2 (12.5)	7 (43.8)	2 (12.5)	2 (12.5)	H = 12.790	*p* = 0.005 **
II (1000–<2000 €)	4 (5.6)	7 (9.7)	28 (38.9)	21 (29.2)	12 (16.7)		
III (2000–<3000 €)	2 (4.2)	12 (25.0)	20 (41.7)	9 (18.8)	5 (10.4)		
IV (≥3000 €)	6 (9.7)	16 (25.8)	28 (45.2)	8 (12.9)	4 (6.5)		
Self-reported health status							
Very good	5 (25.0)	6 (30.0)	9 (45.0)	0 (0.0)	0 (0.0)	H = 40.452	*p* = 0.000 ***
Good	12 (11.4)	25 (23.8)	45 (42.9)	15 (14.3)	8 (7.6)		
Fair	1 (1.0)	11 (11.3)	46 (47.4)	25 (25.8)	14 (14.4)		
Poor	2 (8.7)	0 (0.0)	6 (26.1)	10 (43.5)	5 (21.7)		
Very poor	0 (0.0)	1 (14.3)	2 (28.6)	1 (14.3)	3 (42.9)		
FA Index							
ROBUST	15 (9.2)	34 (20.9)	76 (46.6)	23 (14.1)	15 (9.2)	H = 12.797	*p* = 0.005 **
postROBUST	1 (3.0)	3 (9.1)	15 (45.5)	12 (36.4)	2 (6.1)		
preFRAIL	4 (14.8)	2 (7.4)	10 (37.0)	5 (18.5)	6 (22.2)		
FRAIL	2 (6.1)	4 (12.1)	9 (27.3)	11 (33.3)	7 (21.2)		
Social contacts							
Once or more per week	19 (9.0)	36 (17.1)	91 (43.1)	44 (20.9)	21 (10.0)	H = 4.809	*p* = 0.186
Two or three times per month	2 (10.0)	4 (20)	10 (50.0)	1 (5.0)	3 (15.0)		
Once per month	0 (0.0)	1 (10.0)	5 (50.0)	3 (30.0)	1 (10.0)		
Less than once per month	1 (7.1)	2 (14.3)	3 (21.4)	3 (21.4)	5 (35.7)		
Living in house vs. flat							
Flat	16 (9.5)	29 (17.2)	66 (39.1)	35 (20.7)	23 (13.6)	W = 10893.500	*p* = 0.592
House	6 (6.9)	14 (16.1)	44 (50.6)	16 (18.4)	7 (8.0)		
Objective heat strain							
High	12 (9.3)	22 (17.1)	53 (41.1)	29 (22.5)	13 (10.1)	W = 16461.0	*p* = 0.838
Low	10 (7.9)	21 (16.5)	57 (44.9)	22 (17.3)	17 (13.4)		

**Table 4:** Coping strategies.

Coping Strategy	*n* (%)
Body-related strategies	
Wear less or thinner clothes	256 (99.2)
Drink more fluids	206 (79.8)
Eat differently	195 (75.6)
Shower more frequently	183 (70.9)
Cooling arms with water	78 (30.2)
Using wet towel	60 (23.3)
Cooling feet with water	59 (19.4)
Home-protective strategies	
Open windows for ventilation	250 (96.9)
Use thinner bedding	237 (91.9)
Close blinds/shutters	221 (85.7)
Turn on fan	122 (47.3)
Air Conditioning	10 (3.9)
Activity-related strategies	
Less physical activity	207 (80.2)
Reschedule activities	198 (76.7)

**Table 5:** Body-related coping strategies (Chi^2^-Test, * *p* < 0.05, ** *p* < 0.01, *** *p* < 0.001).

Variables	Body-Related Coping Strategies											
	Drink More Fluids		Eat Differently		Shower More Frequently		Cool Arms with Water		Use Wet Towels		Cool Feet with Water	
	Chi^2^	*p*-Value	Chi^2^	*p*-Value	Chi^2^	*p*-Value	Chi^2^	*p*-Value	Chi^2^	*p*-Value	Chi^2^	*p*-Value
Gender	χ^2^(1) = 2.469	0.116	χ^2^(1) = 2.670	0.102	χ^2^(1) = 0.326	0.568	χ^2^(1) = 7.945	0.005 *	χ^2^(1) = 3.711	0.054	χ^2^(1) = 2.112	0.146
Age classes	χ^2^(2) = 4.117	0.128	χ^2^(2) = 12.096	0.002 **	χ^2^(2) = 6.979	0.031 *	χ^2^(2) = 2.661	0.264	χ^2^(2) = 1.670	0.434	χ^2^(2) = 2.914	0.233
School Leaving Certificate	χ^2^(3) = 8.045	0.045 *	χ^2^(3) = 6.866	0.760	χ^2^(3) = 3.216	0.360	χ^2^(3) = 2.078	0.556	χ^2^(3) = 4.783	0.188	χ^2^(3) = 0.942	0.815
Former Occupation	χ^2^(5) = 9.200	0.101	χ^2^(5) = 7.2740	0.201	χ^2^(5) = 4.115	0.533	χ^2^(5) = 6.577	0.254	χ^2^(5) = 4.233	0.516	χ^2^(5) = 10.563	0.061
Household Income per month	χ^2^(3) = 6.753	0.080	χ^2^(3) = 2.631	0.452	χ^2^(3) = 4.773	0.189	χ^2^(3) = 8.989	0.029 *	χ^2^(3) = 8.042	0.045 *	χ^2^(3) = 5.387	0.146
Health Status	χ^2^(4) = 9.41	0.919	χ^2^(4) = 3.761	0.439	χ^2^(4) = 6.959	0.138	χ^2^(4) = 16.319	0.003 **	χ^2^(4) = 9.210	0.056	χ^2^(4) = 5.339	0.254
Lucas Functional Index	χ^2^(3) = 4.014	0.260	χ^2^(3) = 6.565	0.087	χ^2^(3) = 13.145	0.004 **	χ^2^(3) = 3.284	0.350	χ^2^(3) = 3.497	0.321	χ^2^(3) = 0.924	0.820
GP Consultation	χ^2^(1) = 0.354	0.553	χ^2^(1) = 0.642	0.423	χ^2^(1) = 1.700	0.192	χ^2^(1) = 0.921	0.337	χ^2^(1) = 0.021	0.884	χ^2^(1) = 1.943	0.163
Social Contacts	χ^2^(3) = 7.243	0.065	χ^2^(3) = 0.522	0.907	χ^2^(3) = 3.340	0.342	χ^2^(3) = 3.025	0.388	χ^2^(3) = 2.540	0.468	χ^2^(3) = 0.948	0.814
Household size	χ^2^(2) = 8.086	0.018 *	χ^2^(2) = 4.531	0.104	χ^2^(2) = 3.271	0.195	χ^2^(2) = 2.318	0.314	χ^2^(2) = 1.547	0.461	χ^2^(2) = 0.688	0.709
Objective Heat Strain (area)	χ^2^(1) = 0.507	0.477	χ^2^(1) = 0.228	0.633	χ^2^(1) = 0.047	0.828	χ^2^(1) = 0.036	0.850	χ^2^(1) = 5.241	0.022 *	χ^2^(1) = 0.065	0.800
House or Flat	χ^2^(1) = 4.069	0.044 *	χ^2^(1) = 0.985	0.321	χ^2^(1) = 0.489	0.484	χ^2^(1) = 1.734	0.188	χ^2^(1) = 6.924	0.009	χ^2^(1) = 0.000	0.986

**Table 6:** Home-protective coping strategies (Chi^2^-Test, * *p* < 0.05, ** *p* < 0.01, *** *p* < 0.001).

Variables	Home-Protective Coping Strategies							
	Open Windows		Close Blinds/Shutters		Turn on Fan		Air Conditioning	
	Chi^2^-	*p*-Value	Chi^2^	*p*-Value	Chi-Quadrat	*p*-Value	Chi^2^	*p*-Value
Gender	χ^2^(1) = 1.445	0.229	χ^2^(1) = 0.303	0.582	χ^2^(1) = 0.969	0.325	χ^2^(1) = 0.483	0.487
Age classes	χ^2^(2) = 3.991	0.136	χ^2^(2) = 0.352	0.839	χ^2^(2) = 0.978	0.613	χ^2^(2) = 0.121	0.941
School Leaving Certificate	χ^2^(3) = 2.660	0.447	χ^2^(3) = 0.542	0.910	χ^2^(3) = 2.039	0.564	χ^2^(3) = 3.563	0.313
Former Occupation	χ^2^(5) = 5.162	0.396	χ^2^(5) = 6.465	0.264	χ^2^(5) = 8.567	0.128	χ^2^(5) = 8.000	0.156
Monthly Household Income	χ^2^(3) = 3.871	0.276	χ^2^(3) = 3.610	0.307	χ^2^(3) = 0.879	0.831	χ^2^(3) = 10.067	0.018 *
Health Status	χ^2^(4) = 5.856	0.210	χ^2^(4) = 14.393	0.006 *	χ^2^(4) = 6.458	0.167	χ^2^(4) = 2.395	0.663
Lucas Functional Index	χ^2^(3) = 1.316	0.725	χ^2^(3) = 0.864	0.834	χ^2^(3) = 2.808	0.422	χ^2^(3) = 2.158	0.540
GP Consultation	χ^2^(1) = 0.710	0.399	χ^2^(1) = 2.039	0.153	χ^2^(1) = 0.830	0.362	χ^2^(1) = 0.014	0.906
Social Contacts	χ^2^(3) = 26.456	0.000 ***	χ^2^(3) = 0.685	0.877	χ^2^(3) = 2.618	0.454	χ^2^(3) = 1.675	0.642
Household size	χ^2^(2) = 0.403	0.818	χ^2^(2) = 1.634	0.442	χ^2^(2) = 4.221	0.121	χ^2^(2) = 7.525	0.023 *
Objective Heat Strain (area)	χ^2^(1) = 0.155	0.694	χ^2^(1) = 0.0.239	0.625	χ^2^(1) = 0.014	0.906	χ^2^(1) = 6.788	0.009
House or Flat	χ^2^(1) = 0.103	0.749	χ^2^(1) = 1.931	0.165	χ^2^(1) = 1.472	0.225	χ^2^(1) = 5.963	0.015 *

**Table 7:** Activity-related coping strategies (Chi^2^-Test, * *p* < 0.05, ** *p* < 0.01, *** *p* < 0.001).

Variables	Activity-Related Coping Strategies			
	Reduce Physical Activity		Reschedule Activities	
	Chi^2^-	*p*-Value	Chi^2^-	*p*-Value
Gender	χ^2^(1) = 1.677	0.195	χ^2^(1) = 0.974	0.324
Age classes	χ^2^(2) = 0.612	0.736	χ^2^(2) = 3.756	0.153
School Leaving Certificate	χ^2^(3) = 1.975	0.578	χ^2^(3) = 0.992	0.803
Former Occupation	χ^2^(5) = 4.412	0.492	χ^2^(5) = 2.017	0.847
Household Income per month	χ^2^(3) = 2.912	0.405	χ^2^(3) = 5.130	0.162
Health Status	χ^2^(4) = 7.905	0.095	χ^2^(4) = 1.857	0.762
Lucas Functional Index	χ^2^(3) = 6.555	0.088	χ^2^(3) = 6.320	0.097
GP Consultation	χ^2^(1) = 0.054	0.816	χ^2^(1) = 0.255	0.614
Social Contacts	χ^2^(3) = 0.726	0.867	χ^2^(3) = 0.336	0.953
Household size	χ^2^(2) = 0.422	0.810	χ^2^(2) = 2.273	0.321
Objective Heat Strain (area)	χ^2^(1) = 0.173	0.678	χ^2^(1) = 0.732	0.392
House or Flat	χ^2^(1) = 0.380	0.537	χ^2^(1) = 1.543	0.214

## Data Availability

Not applicable.

## Supplementary Materials

The following are available online at https://www.mdpi.com/article/10.3390/ijerph18147495/s1, Word S1: Kemen_Questionnaire.

## References

[1] World Meteorological Organization (2020). State of the Global Climate 2020.

[2] Muthers S., Laschewski G., Matzarakis A. (2017). The Summers 2003 and 2015 in South-West Germany: Heat Waves and Heat-Related Mortality in the Context of Climate Change. Atmosphere.

[3] Fenner D., Holtmann A., Krug A., Scherer D. (2019). Heat waves in Berlin and Potsdam, Germany—Long-term trends and comparison of heat wave definitions from 1893 to 2017. Int. J. Climatol..

[4] Vautard R., van Aalst M., Boucher O., Drouin A., Haustein K., Kreienkamp F., van Oldenborgh G.J., Otto F.E.L., Ribes A., Robin Y. (2020). Human contribution to the record-breaking June and July 2019 heatwaves in Western Europe. Environ. Res. Lett..

[5] Deutscher Wetterdienst (DWD) (2019). Hitzewelle Juli 2019 in Westeuropa—Neuer Nationaler Rekord in Deutschland.

[6] Deutscher Wetterdienst (DWD) (2009). Deutschlandwetter im Jahr 2020.

[7] World Economic Forum (2020). The Global Risks Report 2020.

[8] Watts N., Amann M., Arnell N., Ayeb-Karlsson S., Beagley J., Belesova K., Boykoff M., Byass P., Cai W., Camp-bell-Lendrum D. (2020). The 2020 report of The Lancet Countdown on health and climate change: Responding to converging crises. Lancet.

[9] Chambers J. (2020). Global and cross-country analysis of exposure of vulnerable populations to heatwaves from 1980 to 2018. Clim. Chang..

[10] United Nations (2015). World Population Ageing 2015.

[11] Divo M.J., Martinez C.H., Mannino D.M. (2014). Ageing and the epidemiology of multimorbidity. Eur. Respir. J..

[12] Mücke H.-G., Litvinovitch J.M. (2020). Heat Extremes, Public Health Impacts, and Adaptation Policy in Germany. Int. J. Environ. Res. Public Health.

[13] Buscail C., Upegui E., Viel J.-F. (2012). Mapping heatwave health risk at the community level for public health action. Int. J. Health Geogr..

[14] Kovats R.S., Johnson H., Griffith C. (2006). Mortality in southern England during the 2003 heat wave by place of death. Health Stat. Q..

[15] Robine J.-M., Cheung S.L.K., Le Roy S., van Oyen H., Griffiths C., Michel J.-P., Herrmann F.R. (2008). Death toll exceeded 70,000 in Europe during the summer of 2003. C. R. Biol..

[16] Bunz M., Mücke H.-G. (2017). Klimawandel—Physische und psychische Folgen. Bundesgesundheitsblatt.

[17] Schifano P., Cappai G., de Sario M., Michelozzi P., Marino C., Bargagli A.M., Perucci C.A. (2009). Susceptibility to heat wave-related mortality: A follow-up study of a cohort of elderly in Rome. Environ. Health.

[18] Stafoggia M., Forastiere F., Agostini D., Biggeri A., Bisanti L., Cadum E., Caranci N., de’Donato F., de Lisio S., de Maria M. (2006). Vulnerability to heat-related mortality: A multicity, population-based, case-crossover analysis. Epidemiology.

[19] Bell M.L., O’Neill M.S., Ranjit N., Borja-Aburto V.H., Cifuentes L.A., Gouveia N.C. (2008). Vulnerability to heat-related mortality in Latin America: A case-crossover study in Sao Paulo, Brazil, Santiago, Chile and Mexico City, Mexico. Int. J. Epidemiol..

[20] Ishigami A., Hajat S., Kovats R.S., Bisanti L., Rognoni M., Russo A., Paldy A. (2008). An ecological time-series study of heat-related mortality in three European cities. Environ. Health.

[21] Naughton M., Henderson A.K., Mirabelli M.C., Kaiser R., Wilhelm J.L., Kieszak S., Rubin C.H., McGeehin M.A. (2002). Heat-related mortality during a 1999 heat wave in Chicago. Am. J. Prev. Med..

[22] Ren C., Williams G.M., Mengersen K., Morawska L., Tong S. (2008). Does temperature modify short-term effects of ozone on total mortality in 60 large eastern US communities? An assessment using the NMMAPS data. Environ. Int..

[23] Zhang Y., Nitschke M., Krackowizer A., Dear K., Pisaniello D., Weinstein P., Tucker G., Shakib S., Bi P. (2017). Risk factors for deaths during the 2009 heat wave in Adelaide, Australia: A matched case-control study. Int. J. Biometeorol..

[24] Curriero F.C. (2002). Temperature and Mortality in 11 Cities of the Eastern United States. Am. J. Epidemiol..

[25] Gouveia N., Hajat S., Armstrong B. (2003). Socioeconomic differentials in the temperature-mortality relationship in São Paulo, Brazil. Int. J. Epidemiol..

[26] Basu R., Ostro B.D. (2008). A multicounty analysis identifying the populations vulnerable to mortality associated with high ambient temperature in California. Am. J. Epidemiol..

[27] Ciancio B., Di Renzi M., Binkin N., Perra A., Prato R., Bella A., Niutta P., Rossi F., Germinario C. (2007). Risk factors for mortality during a heat-wave in Bari (Italy), summer 2005. Ig. E Sanita Pubblica.

[28] Semenza J.C., Rubin C.H., Falter K.H., Selanikio J.D., Flanders W.D., Howe H.L., Wilhelm J.L. (1996). Heat-related deaths during the July 1995 heat wave in Chicago. N. Engl. J. Med..

[29] Mirchandani H.G., McDonald G., Hood I.C., Fonseca C. (1996). Heat-related deaths in Philadelphia—1993. Am. J. Forensic Med. Pathol..

[30] Vandentorren S., Bretin P., Zeghnoun A., Mandereau-Bruno L., Croisier A., Cochet C., Ribéron J., Siberan I., Declercq B., Ledrans M. (2006). August 2003 heat wave in France: Risk factors for death of elderly people living at home. Eur. J. Public Health.

[31] Basu R., Samet J.M. (2002). Relation between Elevated Ambient Temperature and Mortality: A Review of the Epidemiologic Evidence. Epidemiol. Rev..

[32] Bouchama A., Dehbi M., Mohamed G., Matthies F., Shoukri M., Menne B. (2007). Prognostic factors in heat wave related deaths: A meta-analysis. Arch. Intern. Med..

[33] United Nations (2018). World Urbanization Prospects: The 2018 Revision. https://www.un.org/development/desa/en/news/population/2018-revision-of-world-urbanization-prospects.html.

[34] Statistisches Bundesamt (2019). Statistisches Bundesamt. Statistisches Jahrbuch: Deutschland und Internationales 2019.

[35] European Investment Bank The EIB Climate Survey 2019–2020. https://www.eib.org/en/publications/the-eib-climate-survey-2019-2020.htm.

[36] Die Bundesregierung (2008). Deutsche Anpassungsstrategie an den Klimawandel.

[37] Lane K., Wheeler K., Charles-Guzman K., Ahmed M., Blum M., Gregory K., Graber N., Clark N., Matte T. (2013). Extreme heat awareness and protective behaviors in New York City. J. Urban Health.

[38] Akompab D.A., Bi P., Williams S., Saniotis A., Walker I.A., Augoustinos M. (2013). Engaging stakeholders in an adaptation process: Governance and institutional arrangements in heat-health policy development in Adelaide, Australia. Mitig. Adapt. Strat. Glob. Chang..

[39] Liu T., Xu Y.J., Zhang Y.H., Yan Q.H., Song X.L., Xie H.Y., Luo Y., Rutherford S., Chu C., Lin H.L. (2013). Associations between risk perception, spontaneous adaptation behavior to heat waves and heatstroke in Guangdong province, China. BMC Public Health.

[40] Howe P.D., Marlon J.R., Wang X., Leiserowitz A. (2019). Public perceptions of the health risks of extreme heat across US states, counties, and neighborhoods. Proc. Natl. Acad. Sci. USA.

[41] Banwell C., Dixon J., Bambrick H., Edwards F., Kjellström T. (2012). Socio-cultural reflections on heat in Australia with implications for health and climate change adaptation. Glob. Health Action.

[42] Kondo M., Ono M., Nakazawa K., Kayaba M., Minakuchi E., Sugimoto K., Honda Y. (2013). Population at high-risk of indoor heatstroke: The usage of cooling appliances among urban elderlies in Japan. Environ. Health Prev. Med..

[43] Nitschke M., Krackowizer A., Hansen A.L., Bi P., Tucker G.R. (2017). Heat Health Messages: A Randomized Controlled Trial of a Preventative Messages Tool in the Older Population of South Australia. Int. J. Environ. Res. Public Health.

[44] Lindemann U., Skelton D.A., Oksa J., Beyer N., Rapp K., Becker C., Klenk J. (2017). Social participation and heatrelated behavior in older adults during heat waves and on other days. Z. Gerontol. Geriatr..

[45] Conrad K., Penger S. (2020). “Bei Hitze gehe ich nur raus, wenn es wirklich nötig ist!” Empirische Befunde zum Erleben und Verhalten älterer Menschen bei Hitze und Kälte in der Stadt. Psychother. im Alter.

[46] Stadt K. (2015). Starke Veedel—Starkes Köln. Mitwirken, Zusammenhalten, Zukunft Gestalten: Integriertes Handlungskonzept.

[47] Grothues E., Köllner B., Ptak D., Dalelane C., Deutschländer T., Ertel H., Hafer M., Halbig G., Kesseler-Lauterkorn T., Koch C. (2013). Klimawandelgerechte Metropole Köln: Abschlussbericht. LANUV-Fachbericht 50.

[48] Deutscher Wetterdienst (DWD) Heißer Tag. https://www.dwd.de/DE/service/lexikon/Functions/glossar.html?lv2=101094&lv3=101162.

[49] De Bruin A., Picavet H.S.J., Nossikov A. (1996). Health Interview Surveys: Towards International Harmonization of Methods and Instruments.

[50] Dapp U., Minder C.E., Anders J., Golgert S., von Renteln-Kruse W. (2014). Long-term prediction of changes in health status, frailty, nursing care and mortality in community-dwelling senior citizens—results from the Longitudinal Urban Cohort Ageing Study (LUCAS). BMC Geriatr..

[51] Dapp U., Minder C.E., Golgert S., Klugmann B., Neumann L., von Renteln-Kruse W. (2021). The inter-relationship between depressed mood, functional decline and disability over a 10-year observational period within the Longitudinal Urban Cohort Ageing Study (LUCAS). J. Epidemiol. Community Health.

[52] Neumann L., Dapp U., von Renteln-Kruse W., Minder C.E. (2017). Health Promotion and Preventive Care Intervention for Older Community-Dwelling People: Long-Term Effects of a Randomised Controlled Trial (RCT) within the LUCAS Cohort. J. Nutr. Health Aging.

[53] Abrahamson V., Wolf J., Lorenzoni I., Fenn B., Kovats S., Wilkinson P., Adger W.N., Raine R. (2008). Perceptions of heatwave risks to health: Interview-based study of older people in London and Norwich, UK. J. Public Health.

[54] Watts N., Amann M., Arnell N., Ayeb-Karlsson S., Belesova K., Berry H., Bouley T., Boykoff M., Byass P., Cai W. (2018). The 2018 report of the Lancet Countdown on health and climate change: Shaping the health of nations for centuries to come. Lancet.

[55] Dehghan H., Sartang A. (2015). Validation of perceptual strain index to evaluate the thermal strain in experimental hot conditions. Int. J. Prev. Med..

[56] Kalkstein A.J., Sheridan S.C. (2007). The social impacts of the heat-health watch/warning system in Phoenix, Arizona: Assessing the perceived risk and response of the public. Int. J. Biometeorol..

[57] Nitschke M., Hansen A., Bi P., Pisaniello D., Newbury J., Kitson A., Tucker G., Avery J., Dal Grande E. (2013). Risk factors, health effects and behaviour in older people during extreme heat: A survey in South Australia. Int. J. Environ. Res. Public Health.

[58] Bassil K.L., Cole D.C. (2010). Effectiveness of public health interventions in reducing morbidity and mortality during heat episodes: A structured review. Int. J. Environ. Res. Public Health.

[59] Kemen J. (2021). Heat-Health Action—Illustrating the link between heatwaves and drinking behaviour of older adults. Water Risk.

[60] Arnberger A., Allex B., Eder R., Ebenberger M., Wanka A., Kolland F., Wallner P., Hutter H.-P. (2017). Elderly resident’s uses of and preferences for urban green spaces during heat periods. Urban For. Urban Green..

[61] Lee W.V., Shaman J. (2016). Heat-coping strategies and bedroom thermal satisfaction in New York City. Sci. Total Environ..

[62] Van Loenhout J.A.F., Le Grand A., Duijm F., Greven F., Vink N.M., Hoek G., Zuurbier M. (2016). The effect of high indoor temperatures on self-perceived health of elderly persons. Environ. Res..

[63] Kosatsky T., Dufresne J., Richard L., Renouf A., Gianetti N., Bourbeau J., Julien M., Braidy J., Sauvé C. (2009). Heat Awareness and Response among Montreal Residents with Chronic Cardiac and Pulmonary Disease. Can. J. Public Health.

[64] Farbotko C., Waitt G. (2011). Residential air-conditioning and climate change: Voices of the vulnerable. Health Promot. J. Aust..

[65] Hass A.L., Ellis K.N. (2019). Motivation for Heat Adaption: How Perception and Exposure Affect Individual Behaviors during Hot Weather in Knoxville, Tennessee. Atmosphere.

[66] Saliba D., Elliott M., Rubenstein L.Z., Solomon D.H., Young R.T., Kamberg C.J., Roth C., MacLean C.H., Shekelle P.G., Sloss E.M. (2001). The Vulnerable Elders Survey: A tool for identifying vulnerable older people in the community. J. Am. Geriatr. Soc..

[67] Guralnik J.M., Simonsick E.M., Ferrucci L., Glynn R.J., Berkman L.F., Blazer D.G., Scherr P.A., Wallace R.B. (1994). A short physical performance battery assessing lower extremity function: Association with self-reported disability and prediction of mortality and nursing home admission. J. Gerontol..

[68] Umweltbundesamt Der Hitzeknigge. https://www.umweltbundesamt.de/en/publikationen/hitzeknigge.

[69] Lindemann U., Becker C., Roigk P. (2019). Alter und Hitze: Tipps zur Vermeidung von Gesundheitlichen Schäden.

[70] Hass A.L., Ellis K.N. (2019). Using wearable sensors to assess how a heatwave affects individual heat exposure, perceptions, and adaption methods. Int. J. Biometeorol..

[71] Mees H.L.P., Driessen P.P.J., Runhaar H.A.C. (2015). “Cool” governance of a “hot” climate issue: Public and private responsibilities for the protection of vulnerable citizens against extreme heat. Reg. Environ. Chang..

