# Supplementary material for: Heat Perception and Coping Strategies: A Structured Interview-Based Study of Elderly People in Cologne, Germany
Source: Int J Environ Res Public Health. 2021 Jul 14;18(14):7495. doi: 10.3390/ijerph18147495 (PMC8304511; doi:10.3390/ijerph18147495)
Supplement: Supplementary file 1 [file ijerph-18-07495-s001.zip › ijerph-1225099-supplementary.pdf]

Gefördert durch:
Bundesministerium für Umwelt, Naturschutz und nukleare Sicherheit
aufgrund eines Beschlusses des Deutschen Bundestages

HITZE AKTIONS PLAN

RheinEnergie UNIVERSITÄT BONN ihph GeoHealth Centre Stadt Köln

Interviewer ID:

District:

Gefördert durch:

Bundesministerium
für Umwelt, Naturschutz
und nukleare Sicherheit

aufgrund eines Beschlusses
des Deutschen Bundestages

Questionnaire

Heat-Health Action Plan for elderly people in Cologne

Institute for Hygiene and Public Health at the University of Bonn
Environment and Consumer Protection Office of the City of Cologne
Health Department of the City of Cologne
RheinEnergie AG

Contact person for the survey:

Institute for Hygiene and Public Health (IHPH)
University Hospital Bonn
Venusberg-Campus 1
53127 Bonn

Juliane Kemen, IHPH, juliane.kemen@ukbonn.de Tel. +49 228-287-19782
Dr. Silvia Schäffer-Gemein, IHPH, silvia.schaeffer@ukbonn.de, Tel. +49 228-287-14886

Environment and Consumer Protection Office, City of Cologne
Townhouse Deutz – West Building
Willy-Brandt-Platz 2, 50679 Köln

Johanna Grünewald, johanna.gruenewald@stadt-koeln.de
Tel.: +49 221 221 35764

Instructions for completing the questionnaire

For most of the questions, all you have to do is tick one of the boxes.	☒ Yes	☐ No
For some questions multiple answers are possible. Then you are allowed to tick several boxes.	☒ Car	○ Transportation
	○ Bike	☒ by foot
For some questions you will be asked to phrase something in your own words. Please write legibly and clearly.	because it is so hot.	
Please answer the questions in the order in which they are intended. Do not skip questions unless indicated by an arrow.	☒ Yes→ **Question 33**	☐ No

To participate in the survey in writing, please return the completed questionnaire and the signed declaration of consent in the pre-paid envelope to us. The address is written on the envelope.

If you have any questions about the survey or the heat action plan project, please do not hesitate to contact us.

Questionnaire Heat-Health Action Plan for elderly people in Cologne

Thank you for taking part in our survey which is part of the Heat-Health Action Plan project. This survey is about your experiences and your behavior on hot days. In total, it takes about 20-25 minutes to fill out. Please answer all questions in the given order.

Please consider the hot days during this summer for all questions. When we talk about **hot days** in the questionnaire, we mean days with a maximum temperature **over 30 °C.**

As **heat waves** we consider several hot days in a row and it does not cool down much at night.

We will start right away with the first questions about your experience with heat and heat warnings.

A. Heat warnings and Information

**A1. Have you ever read or heard of a heat warning this year?**	
☐ Yes	☐ No → **Question A3**
☐ Don't know	

**A2. What media do you use to read or hear about a heat warning?** Multiple answers possible
O Newspaper or magazine, which ones:
O Website, which ones:
O By Email, which addressor:
O Radio, which radio station:
O TV, which TV channel:
O From friends/relative:
O Via an app:
O Elsewhere:
O Don't know

A3. **What media would you like to receive heat warnings through?** Multiple answers possible
O Newspaper or magazine
O Website
O By Email
O Radio
O TV
O Via an App
O Another medium:_____________
O Don't know

**A4. Do you know the heat warning system of the German Weather Service?**	
☐ Yes	☐ No
☐ Don't know	

B. Heat strain

The following questions are about exposure to heat in your neighborhood and in Cologne.

**B1. On a scale of 1 – no heat impact until 5 – very strong heat impact, how is the heat impact of your city part compared to the rest of the city?**				
1 No heat impact	2 Little heat impact	3 Moderate heat impact	4 Clear heat impact	5 Strong heat impact
☐	☐	☐	☐	☐
☐ Don't know				

**B2. Do you think there is an increase of hot days in Cologne?** This is about your subjective feeling within the period in which you can judge it.	
☐ Yes	☐ No
☐ Don't know	

**B3. Do you think there is an increase of heatwaves in Cologne?** This is about your subjective feeling within the period in which you can judge it.	
☐ Yes	☐ No
☐ Don't know	

HITZE AKTIONS PLAN

B4. Which groups of people do you think are risk groups related to heat?
☐ Don't know

B5. Which people or organizations could you turn to for assistance during heatwaves?

B6. Would you like to get support from the city of Cologne?	
☐ Yes	☐ No → **Question C1**
☐ Don't know	

B7. What kind of support would you like to get from the city of Cologne during heatwaves?

C. Heat coping

The following questions are about arrangements you can carry out to adapt your home or yourself during a heatwave.

**C1. What do you do during an episode of heat to protect yourself?**<br>Simply phrased: What do you do when it is hot?

Now we are going to ask you some questions about certain behaviors.

**C2. Do you open the windows of your apartment / house for ventilation?**	
☐ Yes → **Question C4**	☐ No
☐ Don't know	

**C3. Why don't you open the windows for ventilation?**

**C4. When do you open your windows for ventilation?**<br>Multiple answers possible				
O In the morning	O In the evening	O At noon	O All day	O At night
O Different time:			O Don't know	

**C5. What options do you have for insulation or external shading in your house/apartment?**<br>Multiple answers possible		
O Curtains	O Shutters	O Awning
O Window shutters	O Insulation of the roof	O Partial insulation of the house
O Insulation of the whole house	O None of the above	O Others:
O Don't know		

C6. Do you use curtains, shutters, window shutters and / or awnings to protect yourself against heat?	
☐ Yes	☐ No
☐ Don't know	

C7. Do you use a fan?	
☐ Yes	☐ No → **Question C9**
☐ Don't know	

C8. Do you open the window while using the fan?	
☐ Yes	☐ No
☐ Don't know	

C9. Do you have air conditioning?	
☐ Yes	☐ No → **Question C12**
☐ Don't know	

C10. What temperature do you set the air conditioning to?
☐ Don't know

C11. How often do you use the air conditioning on hot days?<br>Multiple answers possible			
O The whole day	O Several hours a day	O Very rarely	O Never
O Don't know			

C12. Do you wear lighter clothes on hot days?	
☐ Yes	☐ No
☐ Don't know	

C13. Do you use thinner bedding on hot days?	
☐ Yes	☐ No
☐ Don't know	

**C14. Do you use water to cool yourself?**<br>Multiple answers possible	
O Yes, by showering or taking a cool bath	O Yes, with wet towels
O Yes, by cooling arms with water	O Yes, by cooling feet with water
O No	O Don't know

**C15. Do you move less and/or take more breaks?**	
☐ Yes	☐ No
☐ Don't know	

**C16. Do you reschedule activities during hot days?**	
☐ Yes	☐ No → **Question C25**
☐ Don't know → **Question C25**	

**C17.Which activity do you reschedule during hot days?**<br>Enter here the first activity you postpone. Enter further activities in the following questions.

**C18. To what time of day do you postpone the activity?**		
☐ To the evening hours	☐ To the morning hours	☐ Different period of time:
☐ Don't know		

**C19. Do you reschedule a second activity during hot days?**	
☐ Yes	☐ No<br>→ **Question C25**
☐ Don't know → **Question C25**	

**C20. Which second activity do you reschedule during hot days?**<br>Enter here the second activity you postpone.

C21. To what time of day do you postpone the second activity?		
☐ To the evening hours	☐ To the morning hours	☐ Different period of time:
☐ Don't know		

C22. Do you reschedule a third activity during hot days?	
☐ Yes	☐ No<br>→ **Question C25**
☐ Don't know→ **Question C25**	

C23. Which third activity do you reschedule during hot days?<br>Enter here the third activity you postpone.

C24. To what time of day do you postpone the third activity?		
O To the evening hours	O To the morning hours	O Different period of time:
O Don't know		

C25. Imagine a day of around 20 degrees outside. How much do you drink in total on such a day?<br>You can state this either in glasses or in liters. One glass holds 0.2 liters.	
___________ glasses	____________ liters
☐ Don't know	

C26. What kind of beverages do you usually drink on a normal day?		
O Tap water	O Mineral water	O Coffee
O Tea	O Juice	O Other drinks:
O Don't know		

C27. Do you drink more fluids on hot days and if so, how much do you drink in total?	
☐ Yes, about _________ glasses → **Question C29**	☐ Yes, about _________ liters → **Question C29**
☐ No → **Question C28**	
☐ Don't know	

**C28. You have stated that you do not drink more fluids than usual when it is hot. What are your reasons?** Multiple answers possible	
O Not thirsty	O Different reasons:
O Don't know	

**C29. How many portions of fruit do you usually eat per day?** One portion fits in a hand.				
☐ None	☐ 1 portion	☐ 2 portions	☐ 3 portions	☐ 4 or more portions
☐ Don't know				

**C30. How many portions of vegetables do you usually eat per day?** One portion fits in a hand.				
☐ None	☐ 1 portions	☐ 2 portions	☐ 3 portions	☐ 4 or more portions
☐ Don't know				

**C31. Do you eat differently than usual on hot days?**	
☐ Yes	☐ No → **Question D1**
☐ Don't know → **Question D1**	

**C32. You stated that you eat differently on hot days. How are you changing your diet?**

D. Mobility

D1. Is it warmer inside your apartment / house than outside on hot days?		
☐ Yes, mostly/always	☐ Partly	☐ No
☐ Don’t know		

D2. Do you leave your apartment/house on hot days?			
☐ Yes, more often than usual.	☐ Yes, just as often as usual. → **Question D4**	☐ Yes, less than usual.	☐ No, I avoid going outside.
☐ Don’t know			

D3. Why do you leave your apartment/house more, less often or not at all?

D4. Which of the following locations would you like to visit during hot days?<br>Multiple answers possible
O Cooler rooms inside the house
O Cooler rooms outside the house
O Parks and green spaces
O Greenspace and nature areas with water such as the Rhine river, streams or lakes
O Don’t know

D5. What challenges or difficulties do you experience on hot days on your everyday journeys?<br>For example, a lack of shady paths
☐ Don’t know

E. Social network

E1. How often do you usually meet other people?			
☐ Once to several times a week.	☐ Two to three times a month.	☐ Once a month.	☐ Less often or never.
☐ Don't know			

E2. Does the number of people you meet change during heat waves?		
☐ Yes, I meet other people more often.	☐ Yes, I meet other people less often.	☐ No
☐ Don't know		

E3. Do you know someone who would take care of you for a few days if necessary?<br>This means groups of people such as partners, relatives, acquaintances or neighbors.<br>Do not enter names and addresses here, but only a group of persons.	
☐ Yes:	☐ No
☐ Don't know	

F. Health effects of heat

F1. On a scale from 1 – no heat strain at all to 5 – extreme heat strain, how would you rate your personal heat strain?				
1<br>Not heat strain at all	2<br>Little heat strain	3<br>Moderate heat strain	4<br>Clear heat strain	5<br>Extreme heat strain
☐	☐	☐	☐	☐
**→ Question F2**	**→ Question F2**	**→ Question F3**	**→ Question F3**	**→ Question F3**
☐ Don't know				

F2. You stated that you feel no or little heat strain. What are your reasons for this answer?

F3. You stated that you feel moderate to extreme heat strain. What are your reasons for this answer?

F4. How would you rate your risk for personal health issues during heatwaves, on a scale from 1 - very low to 5 - very high?

1<br>Very low	2<br>Low	3<br>Medium	4<br>High	5<br>Very high
☐	☐	☐	☐	☐

☐ Don't know

F5. Do you experience fear of heat?

☐ Yes	☐ No → **Question F7**
☐ Don't know → **Question F7**	

F6. What exactly are you afraid of?

The following is about symptoms or illnesses that can result from heat.

F7. What heat symptoms or illnesses did you experience during heat waves this summer?

F8. Have you experienced the following symptoms or illnesses caused by heat this summer?			
Sleeping disorder	O Yes	O No	O Don't know
Fatigue	O Yes	O No	O Don't know
Anxiousness	O Yes	O No	O Don't know
Concentration issues	O Yes	O No	O Don't know
Headache	O Yes	O No	O Don't know
Immense thirst	O Yes	O No	O Don't know
Extraordinary sweating	O Yes	O No	O Don't know
Dehydration	O Yes	O No	O Don't know
Dizziness	O Yes	O No	O Don't know
Sunburn	O Yes	O No	O Don't know
Nausea	O Yes	O No	O Don't know
Vomitting	O Yes	O No	O Don't know
Diagnosed sunstroke	O Yes	O No	O Don't know
Diagnosed heat stroke	O Yes	O No	O Don't know

F9. Has your general practitioner (GP) ever talked to you about heat coping strategies?	
☐ Yes	☐ No → **Question F11**
☐ Don't know	

F10. Do you remember what he / she told you?

F11. Has your GP ever talked to you about heat in view of your health condition?	
☐ Yes	☐ No → **Question F13**
☐ Don't know	

F12. Do you remember what he / she told you?

HITZE AKTIONS PLAN

**F13. Has your GP ever talked to you about heat in view of your medication(s)?**	
☐ Yes	☐ No → **Question F15**
☐ Don't know	

**F14. Do you remember what he / she told you?**

**F15. Has your GP ever talked to you about who could take care of you if you are not doing well during heat waves?**	
☐ Yes	☐ No
☐ Don't know	

**F16. How many minutes do you need to get to your GP?**
__________ minutes
☐ Don't know

**F17. What means of transport do you usually use to get to your GP?** Multiple answers possible
O Walking
O By bike
O With public transport
O By car
O Different transport:
O Don't know

G. General health

G1. How is your health in general from 1 – very good bis 5 – very poor?<br>Reference: [22]				
1<br>very good	2<br>good	3<br>fair	4<br>poor	5<br>very poor
☐	☐	☐	☐	☐
☐ Don't know				

G2. Do you need daily assistance by medical professionals (Medical Service Level)?	
☐ Yes	☐ No → **Question G4**
☐ Don't know → **Question G4**	

G3. To which medical service level are you classified?
☐ Service Level 1
☐ Service Level 2
☐ Service Level 3
☐ Service Level 4
☐ Service Level 5
☐ Don't know

G4. Have you lost 5 kg (a stone) or more over the past 6 months without trying to do so?<br>Reference: [23]	
☐ Yes	☐ No
☐ Don't know	

G5. In the past 12 months, has your health had an impact on:<br>Reference: [23]			
**... your ability to walk up to 1 kilometer in distance?**	☐ Yes	☐ No	☐ Don't know
**... your ability to climb 10 steps?**	☐ Yes	☐ No	☐ Don't know
**... your ability to get into or out of a car or bus?**	☐ Yes	☐ No	☐ Don't know

G6. Over the past 7 days, how often did you take a walk outside your home or garden for any reason?<br>Reference: [23]
☐ Never
☐ 1-2 days per week
☐ 3-4 days per week
☐ 5-7 days per week
☐ Don't know

G7. During the 12 past months, have you ever fallen to the ground or floor?<br>Reference: [23]	
☐ Yes	☐ No
☐ Don't know	

G8. Are you able to walk 500 meters?<br>Reference: [23]
☐ Yes, without difficulties
☐ Yes, with difficulties
☐ Yes, with a device
☐ Yes, with help from someone
☐ No, not possible
☐ Don't know

G9. Over the past 7 days, how often did you engage in strenuous sport or recreational activities?<br>Reference: [23]
☐ Never
☐ 1-2 days per week
☐ 3-4 days per week
☐ 5-7 days per week
☐ Don't know

G10. Over the past 7 days, how often did you engage in moderate sport or recreational activities?<br>Reference: [23]
☐ Never
☐ 1-2 days per week
☐ 3-4 days per week
☐ 5-7 days per week
☐ Don't know

G11. Do you work as a volunteer?<br>Reference: [23]		
☐ No	☐ Yes, part-time	☐ Yes, full-time
☐ Don't know		

G12. Do you limit your activities because you are afraid you will fall?<br>Reference: [23]	
☐ Yes	☐ No
☐ Don't know	

G13. How tall are you?

G14. What is your weight in kg, please?

H. Personal and household characteristics

H1. Please indicate your school leaving certificate.
☐ Academic secondary school (Abitur)
☐ Secondary school
☐ Secondary general school
☐ Domestic economy school (Hauswirtschaftsschule)
☐ No school certificate
☐ Another school leaving certificate:
☐ Don't know

H2. Which kind of educational training did you attend after school?
☐ Academic degree
☐ Apprenticeship
☐ No further educational training
☐ Don't know

HITZE AKTIONS PLAN

**H3. What is your occupation today?**<br>Multiple answers possible
☐ On pension/retirement pay → **Question H5**
☐ Working → **Question H4**
☐ Different: → **Question H5**
☐ Don't know → **Question H5**

**H4. How many hours do you currently work each week /each day?**
________________ hours

**H5. To which professional occupation group did you belong to before retirement?**
☐ Self-employed
☐ Employee or public servant in a managing position
☐ Employee or public servant
☐ Skilled worker
☐ Worker
☐ Farmer
☐ Housewife/homemaker
☐ Don't know

**H6. In which job did you work?**

**H7. Could you please tell us your age?**
______ years

**H8. And your gender?**		
☐ Female	☐ Male	☐ Divers

**H9. Do you live…**
☐ alone
☐ with one other person
☐ with more than one person
☐ Don't know

H10. How many persons, including yourself, live in your household?
______________ persons

H11. Do you live in a flat or house?	
☐ flat	☐ house

H12. How many square meters is your flat or house?
☐ Don’t know

H13. How much is the monthly income of your household?
☐ Less than 500€
☐ 500€ to <1000€
☐ 1000€ to <2000€
☐ 2000€ to <3000€
☐ More than 3000€
☐ Don’t know

H14. Do you want to tell us anything which comes to your mind regarding the topic of heat? What is important for you?

Thank you very much for participating!
